# When microscopy and electrophysiology meet connectomics—Steve Massey’s contribution to unraveling the structure and function of the rod/cone gap junction

**DOI:** 10.3389/fopht.2023.1305131

**Published:** 2023-11-17

**Authors:** Christophe P. Ribelayga, John O’Brien

**Affiliations:** Department of Vision Sciences, University of Houston College of Optometry, Houston, TX, United States

**Keywords:** gap junction, dopamine, melatonin, electrical synapse coupling, retina

## Abstract

Electrical synapses, formed of gap junctions, are ubiquitous components of the central nervous system (CNS) that shape neuronal circuit connectivity and dynamics. In the retina, electrical synapses can create a circuit, control the signal-to-noise ratio in individual neurons, and support the coordinated neuronal firing of ganglion cells, hence, regulating signal processing at the network, single-cell, and dendritic level. We, the authors, and Steve Massey have had a long interest in gap junctions in retinal circuits, in general, and in the network of photoreceptors, in particular. Our combined efforts, based on a wide array of techniques of molecular biology, microscopy, and electrophysiology, have provided fundamental insights into the molecular structure and properties of the rod/cone gap junction. Yet, a full understanding of how rod/cone coupling controls circuit dynamics necessitates knowing its operating range. It is well established that rod/cone coupling can be greatly reduced or eliminated by bright-light adaptation or pharmacological treatment; however, the upper end of its dynamic range has long remained elusive. This held true until Steve Massey’s recent interest for connectomics led to the development of a new strategy to assess this issue. The effort proved effective in establishing, with precision, the connectivity rules between rods and cones and estimating the theoretical upper limit of rod/cone electrical coupling. Comparing electrophysiological measurements and morphological data indicates that under pharmacological manipulation, rod/cone coupling can reach the theoretical maximum of its operating range, implying that, under these conditions, all the gap junction channels present at the junctions are open. As such, channel open probability is likely the main determinant of rod/cone coupling that can change momentarily in a time-of-day- and light-dependent manner. In this article we briefly review our current knowledge of the molecular structure of the rod/cone gap junction and of the mechanisms behind its modulation, and we highlight the recent work led by Steve Massey. Steve’s contribution has been critical toward asserting the modulation depth of rod/cone coupling as well as elevating the rod/cone gap junction as one of the most suitable models to examine the role of electrical synapses and their plasticity in neural processing.

## Introduction

Gap junctions are anatomical structures that were discovered around the time that electron microscopy (EM) was introduced, that is in the 1960s (1). The cross-sectional view of the plasma membranes visualized via transmission EM offers detailed views of gap junctions, which typically appear as electron-dense pentalaminar structures of ≈ 10 nm in thickness and where the separation between the membranes of the adjacent cells is ≈ 3 nm. Central to these structures are aggregates of intramembranous particles in the two apposed membranes meeting particle to particle in the ≈ 3-nm intermembrane “gap” (1, 2). These aggregates are transmembrane channels that serve as conduits between the cytoplasm of the two adjacent cells, which in turn allow the diffusion of small molecules, such as electrolytes, secondary messengers, and metabolites (1). In addition, the electron-dense material often extends into the adjacent cytoplasmic areas and includes a variety of scaffolding proteins and regulatory proteins (1, 2). Using the freeze-fracture EM technique, gap junctions typically present as ensembles or clusters of channels that can appear in several different morphological forms and densities, from strings containing a few dozens of channels to large, packed, crystalline plaques containing more than 300 channels (2, 3). Gap junctions between neurons mediate electrical coupling by allowing the direct flow of current between neurons (1, 2). Gap junction-mediated electrical coupling has been observed in many areas of the brain, including the thalamus (4, 5), hippocampus (6, 7), neocortex (8–10), cerebellar cortex (11–15), inferior olive (16), and retina (17–20).

**Figure 1:**
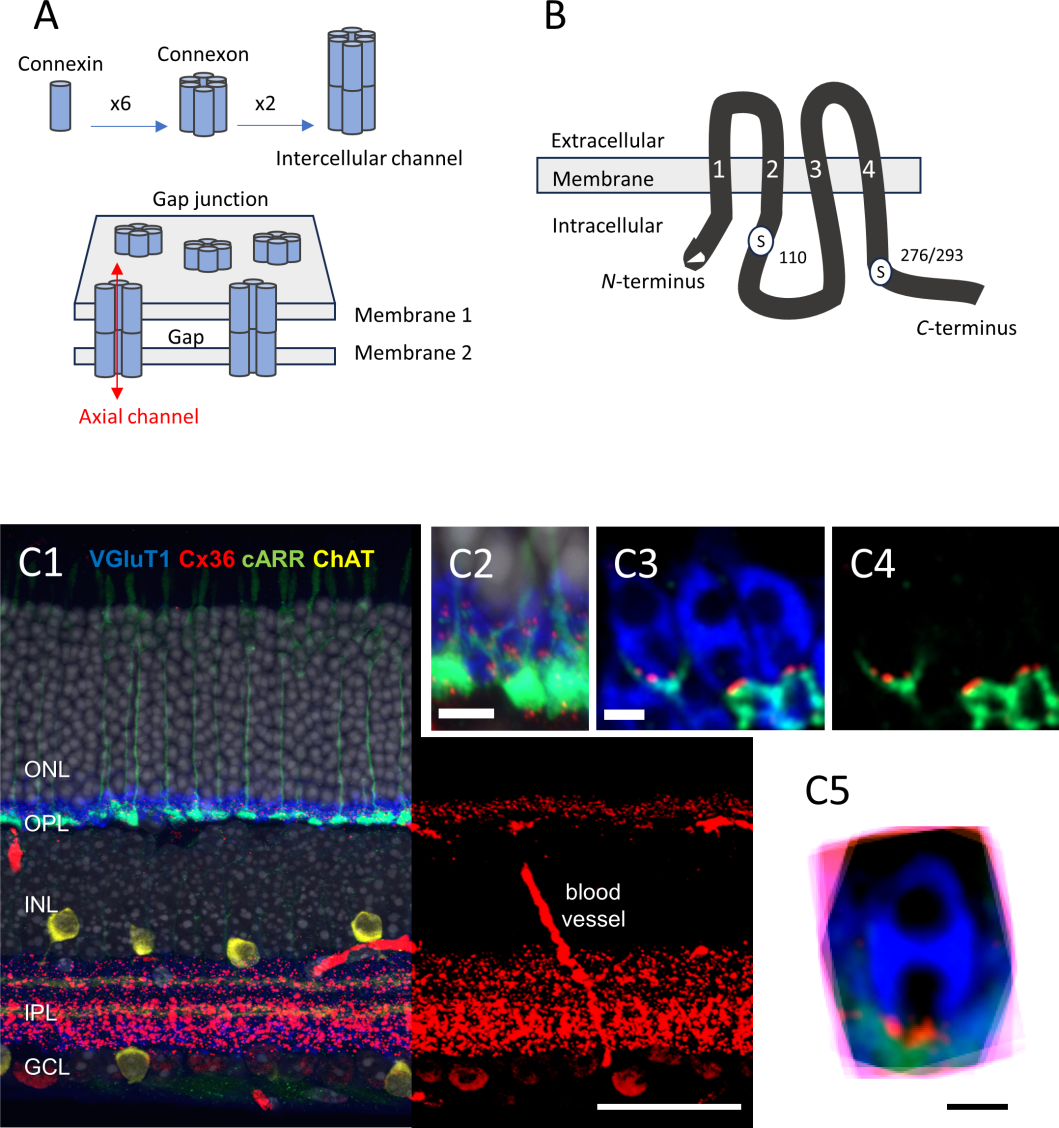
**(A)** Schematic representation of the multiple levels of a gap junction’s molecular structure. The figure is modified from Goodenough and Paul (21); see text for details. **(B)** Diagram showing the molecular structure of Cx36, including the two serine residues that can be phosphorylated, namely S110, and S276 (in teleost)/S293 (in mice). The transmembrane domains are labeled “1” to “4”. The figure is modified from Kothmann et al. (22). **(C1–5)** Immunolocalization of Cx36 in mouse retina. **(C1)** Five-channel labeling of wild-type mouse (B6) retinal sections, which were obtained by confocal microscopy (Zeiss 780). The cones are labeled for cone arrestin (cARR green), the rod spherules for the vesicular glutamate transporter 1 (vGluT1, blue), starburst amacrine cells are labeled for choline acetyltransferase (ChAT, yellow), and the nuclei are stained with 4′,6-diamidino-2-phenylindole (DAPI) (gray). The right part of the retinal section shows only the red channel (Cx36), for clarity. The Cx36 labeling is very dense in the IPL, and less so in the OPL. Scale bar, 50 μm. **(C2)** The image obtained at a higher magnification. Note the cone pedicles, rod spherules, and Cx36 contained in the OPL. Scale bar, 10 μm. **(C3)**. Confocal microscopy with Airyscan. The representative example shows three rod spherules (blue) and cone telodendria (green) with multiple Cx36 puncta (1 to 4, red) at each contact. Scale bar, 1 μm. **(C4)** The same as C3, but with no blue channel for clarity. Scale bar, 1 μm. **(C5)** Eighteen rod spherules, each from one optical section, aligned, and then superimposed. Note that Cx36 clusters (red) are found at the base of the rod spherule, close to the opening of the postsynaptic compartment. Scale bar, 1 μm. Modified from Jin et al. (23) and Ishibashi et al. (24). ONL, outer nuclear layer; OPL, outer plexiform layer; INL, inner nuclear layer; IPL, inner plexiform layer; GCL, ganglion cell layer.

Over the last two decades, scientists have made remarkable progress toward understanding the molecular structure of gap junction channels. Each channel is formed of two hemichannels or connexons, each anchored in one of the apposed cell membranes (**Figure 1A**). The docking, head to head, of the two connexons forms a continuous intercellular pore. Each hemichannel is assembled from six connexins (21). Dozens of different connexins have been found in vertebrates, including 20 in mammals (25). A hemichannel can be composed entirely of one connexin (homomeric) or of different connexins (heteromeric). Also, a gap junction channel can be composed of two hemichannels with the same (homotypic) or different (heterotypic) connexin composition. Of note is that each coupled cell contributes its own connexin(s)/hemichannel(s); if only one cell does, then no gap junction forms and functional coupling is not detected (26). The molecular complexity of single channels and the variation theme in channel clusterization suggest a rich potential for functional diversity. Adding to the complexity of the morphology, electrical synapses are highly dynamic structures, similar to their chemical counterparts (17, 19, 27–33).

The neural retina has emerged as the model system of choice to study electrical coupling in the CNS (17). The well-laminated structure of the retina, the advanced knowledge of its fundamental plan, and the presence of electrical coupling among all five classes of neurons have facilitated the detailed analysis of the distribution, function, and plasticity of gap junctions. When it became clear that most classes of neurons in the retina were electrically coupled, Steve Massey, who was already a renowned anatomist, saw the opportunity. He built a research group whose focus would be electrical synapses in retinal circuits. Steve Mills, who was already a member of the Vision research group, converted to the new cult. John O’Brien who had just cloned the first neuron-specific connexin (34) would join the group and bring his expertise on the molecular structure and plasticity of gap junctions. Christophe P. Ribelayga, who had been studying how rod/cone coupling changes with the time of day, brought expertise in electrophysiology and circadian biology to the group. Although we each had our own individual research program, the influence of Steve Massey in our research was evident. A major focus was the rod/cone gap junction, both individually and collectively. Below we review the work that led to our current understanding of the structure, distribution, plasticity, and function of the rod/cone gap junction. In addition, we review recent work on rod/cone connectivity initiated by Steve and the remarkable results that this work generated.

## Early findings

Early evidence that gap junctions are present between rod and cone photoreceptors was provided by EM studies in the 1970s (35, 36). In rabbit, macaque, turtle, and cat retinas, rod/cone gap junctions were consistently found to be close to the entrance of the invagination of the rod spherule. Using a freeze-fracture EM technique, Raviola and Gilula (35) further showed that the gap junction channels align as strings of single channel thick and about 50 channels long, forming incomplete rings around the mouth of the rod spherules. Electrophysiology experiments subsequently demonstrated that electrical coupling between the rods and cones is functionally relevant—*rod and cone signals can mix* (37–42). Altogether, early work demonstrated that gap junctions are present at rod/cone contacts and that rod/cone coupling is functional.

## Rod/cone gap junctions are made of connexin36

One of the first connexins to be cloned was connexin35 (Cx35), in perch (43) (**Figure 1B**). In the perch retina, immunostaining for Cx35 labels both the outer plexiform layer (OPL) and the inner plexiform layer (IPL) (43). A detailed microscopic analysis in zebrafish OPL showed that Cx35 was present at contacts between the cone pedicles, between the rod spherules, and between the rod spherules and the cone pedicles (rod/cone contacts) (44). The expression pattern in the outer retina appeared to be slightly different in salamander, where Cx35 puncta are also found between rod somata, high in the outer nuclear layer (ONL) (45).

In the mammalian OPL, immunolabeling of Cx36 (the ortholog of Cx35) is primarily found at rod/cone contacts in macaques (46) and mice (47) (**Figure 1C**), whereas it is mostly found at contacts between cones in the cone-dominated ground squirrel (48). The question of whether or not mammalian rods express Cx36 and form rod/rod gap junctions was controversial for some time (see for instance 47, 49, and 50). As it turned out, it appeared to be two separate questions. In mice, rods express Cx36 (51) and Cx36 exclusively (23). The cones express Cx36 exclusively (23) and form gap junctions with adjacent cones at telodendritic contacts, as well as with adjacent rods at contacts typically located between the tip of a telodendritic process and a rod spherule, close to the mouth of that rod spherule (23, 24). Thus, although rods express Cx36 and are densely packed in most mammalian retinas, the presence of gap junctions between rod spherules (i.e., rod/rod gap junctions) remained difficult to ascertain in mouse (47) and macaque retina (46); however, one study did report rod/rod gap junctions in mice using transmission EM (52). In contrast, electrical coupling between mammalian rods has been well documented (53–55). To solve the discrepancy, we created mouse lines in which Cx36 was selectively eliminated in rods or in cones (23). In the cone-specific Cx36 conditional knock-out (cKO) mouse, we expected to eliminate the Cx36 immunosignal between the cones and between the rods and cones while leaving the signal intact, if any, between the rod spherules (23). We found, however, that Cx36 immunosignal was decreased by > 95% in the OPL in the cone-specific Cx36 cKO and that no immunolabeling was clearly present between the rod spherules (23). Yet, consistent with Cx36 being expressed in the rods, electrical coupling between the rods was abolished when Cx36 was selectively eliminated from the rods (rod-specific Cx36 cKO) (23). Unexpectedly, we found that electrical coupling between the rods was also eliminated in the cone-specific Cx36 cKO (23). Altogether, these surprising findings indicate that electrical coupling between mouse rods is not direct (rod/rod or rod-to-rod coupling), but indirect (via rod/cone coupling or rod-to-cone-to-rod coupling) (23). The weak Cx36 immunostaining observed at the cone pedicles in the rod-specific Cx36 cKO suggest that cone/cone coupling is rare in mice (23). Thus, electrical coupling between mammalian photoreceptors is mainly supported by rod/cone gap junctions made of Cx36.

## Rod/cone electrical coupling is tightly regulated by the time of day

Sharp electrode recordings from goldfish cone horizontal cells (cHCs) provided the early evidence that rod/cone coupling changes with the time of day and/or lighting conditions (40). Cone input to cHCs predominates during the day, but rod input predominates at night (40, 56), even though cHCs make chemical synaptic contact exclusively with cones (57). Recording directly from the cones revealed that the rod signals enter the cones and cHCs to a greater extent at night, and, therefore, that rod input in cHCs originates from cones (41). Consistent with the presumed increase in rod/cone coupling at night, tracer coupling between photoreceptors is increased at night in goldfish (41), zebrafish (44), rabbit (58), and mice (41, 47, 54).

Changes in the phosphorylation state of Cx35/36 support changes in electrical coupling. Specifically, phosphorylation of Cx35/36 at serine residues S110 and S276 (in teleosts) or S293 (in mammals) is required to increase tracer coupling *in vitro* (30, 33) (**Figure 1B**). Immunoreactivity of phosphorylated Cx35/36, as assessed by phosphoantibodies against S110 and/or S276/293, is low in the OPL during the day and is dramatically increased at night [44 (zebrafish), 47 (mouse); 59 (mouse)]. The day/night difference in tracer coupling correlated to Cx35/36 phosphorylation state is about 24-fold in both zebrafish (44) and mice (47, 59).

Dopamine is key to the daily changes in rod/cone coupling. Dopamine is released by a unique type of amacrine cell, the dopaminergic amacrine cell (60). Dopamine release is controlled by light and a circadian clock so that dopamine release is high during the day and low at night (61, 62, for reviews). Dopamine acts, via D_2_-like/D_4_ membrane receptors on rods and cones, to modulate rod/cone coupling. These receptors are negatively coupled to the adenylate cyclase/cAMP/protein kinase A pathway. Thus, activation of dopamine receptors and the subsequent decrease in protein kinase A activity decreases Cx35/Cx36 phosphorylation [44 (zebrafish), 2013 (mouse)], tracer coupling between photoreceptors [41 (goldfish and mouse); 44 (zebrafish), 47 (mouse); 54 (mouse)], and eventually rod input to the cones [41 (goldfish); 23 (mouse)] and to second-order cells [56 (goldfish); 63 (rabbit)]. The circadian component of dopamine signaling reflects rhythms in both D_2_-like/D_4_ receptor expression in photoreceptors [47 (mouse)] and dopamine release that is driven by melatonin [64 (goldfish); 65 (mouse)]. Melatonin produced in the retina and whose levels peak at night suppresses dopamine release from dopaminergic amacrine cells (61, 62, for reviews). The result is that rod/cone coupling is stronger at night, when melatonin levels are high and dopamine levels low, than during the day, when melatonin levels are low and dopamine levels high (19, 62). Of note, in many strains of mice, including the common B6 strain, a rhythm of melatonin production is absent or of low amplitude because of genetic mutations in the key enzymes of the melatonin synthetic pathway [aralkylamine *N*-acetyltransferase (AANAT) and acetylserotonin *O*-methyltransferase (ASMT); see 61, 62, for reviews]. Backcrossing B6 mice to a melatonin-proficient mouse strain (i.e., CBA/CaJ) to incorporate the desirable *aanat* and *asmt* genes rescued both rhythms of melatonin synthesis and dopamine release (Zhang et al., 2018). It follows that in the melatonin-deficient mouse strains, the circadian rhythm in rod/cone coupling is of low amplitude, and the daily changes in dopamine release and rod/cone coupling are mainly driven by light and dark (47). Consistent with dopamine signaling being the highest during the day or subjective day, dopamine agonists mimic the daytime state and dopamine antagonists mimic the nighttime state of rod/cone coupling [23 (mouse)]. Other neuromodulators may control rod/cone coupling as well. For instance, adenosine, whose extracellular levels in the retina are increased at night [66 (rabbit); 67 (goldfish)], displays antagonistic actions on rod/cone coupling compared with dopamine [47 (mouse) and 68 (zebrafish)]. Thus, the push–pull modulation supported by dopamine and adenosine results in tight control of rod/cone coupling during the day and night, a process that clearly underlies the functional significance of rod/cone coupling.

## How strong can rod/cone coupling be?

To fully comprehend the functional significance of rod/cone coupling on retinal function, the limits of rod/cone coupling strength must be known. Simultaneous paired recording of the rod/cone transjunctional conductance using a whole-cell patch-clamp technique provided quantitative data on the modulation of rod/cone coupling by dopamine in mouse retina (23, 69). The application of a dopaminergic D_2_-like agonist (e.g., quinpirole) decreased rod/cone coupling to nearly 0 pS (where background noise is 50 pS; 23), a result consistent with the effect of D_2_-like agonists on the phosphorylation state of Cx36 at rod/cone contacts (47), photoreceptor tracer coupling (41, 47, 54), and electrical coupling (54, 55, 69). In contrast, the application of a D_2_-like antagonist (e.g., spiperone) increased rod/cone coupling to about 1,200 pS (23). These results are also consistent with the previously reported effects of D_2_-like antagonists on the phosphorylation state of Cx36 at rod/cone contacts (47), photoreceptor tracer coupling (41, 47, 54), and electrical coupling (54, 55, 69). Interestingly, this ≈ 24-fold range in conductance (1,200/50) is close to those reported for the Cx36 phosphorylation state (≈ 24-fold) and tracer coupling (≈ 24-fold) when measured with dopamine agonists/antagonists in mouse retina (47). The similarity between the measurements indicates a causal relationship. In addition, the fact that a D_2_-like agonist acts rapidly (within 10–15 min; CPR and JOB, personal observations) suggests that dopamine may act through modulating the phosphorylation state of Cx36 channels that are already present at rod/cone contacts, rather than by adding new channels to the existing gap junction. In support of this, neither the number of Cx35/36 puncta nor the mean Cx36 immunosignal in the OPL was found to change between the day and night or between lighting conditions (44, 47, 59), although one study reported a nighttime increase in *Cx36* gene expression in the ONL (70). Yet, whether or not blocking dopamine receptors with a D_2_-like antagonist pushes the rod/cone conductance to the maximum has long remained an important unanswered question. The fraction of open channels or the open probability of a channel is usually considered to be very low at electrical synapses (i.e., < 1%; 71, 72), although values of up to 18% have been reported for Cx36 gap junctions in mouse cerebellum (15). To determine the fraction of open channels at a specific electrical synapse, one needs to know how many channels there are in the first place. The product of the number of channels by the unitary conductance yields the theoretical maximum conductance; the ratio of the measured conductance by the maximal theoretical conductance gives the open probability of a single channel. To generalize the results, one also needs to determine the connectivity pattern and determine whether this is a repeated pattern and correct for possible divergence/convergence. Answering these important questions in the case of rod/cone gap junctions necessitated establishing the rules of connectivity between the rods and cones and the average size of rod/cone gap junctions from large datasets. Steve Massey was up to the challenge. The approach was based on combining different techniques of microscopy: confocal microscopy, serial block face scanning EM (SBF-SEM), and focused ion beam scanning EM (FIB-SEM) (24).

### Confocal microscopy

Confocal microscopy with improved resolution (Airyscan) was used to localize Cx36 in the OPL. Immunofluorescent labeling for Cx36 revealed small clusters of labeling at rod/cone contacts (**Figure 1C**). Specifically, these potential gap junctions are located at contacts between the fine processes (telodendria), which emanate from the cone pedicles and extend laterally from each cone pedicle to form an overlapping matrix in the OPL. The cone telodendria also rise up above the level of the cone pedicles to contact the overlying rod spherules. As the complexity of the matrix of telodendritic processes made it difficult to analyze individual cone pedicles with confidence, sparse genetic labeling of a few individual cones was used. The mean number of puncta per cone pedicle was found to be approximately 51. The combination of confocal and genetic approaches was also effective to establish that blue (S) cones are connected to rods in a manner indistinguishable from the green (M) cones. Finally, more than one Cx36-labeled point was observed on rod spherules (2.5 on average). Altogether, and presuming that a Cx36 cluster indicates the presence of a gap junction, the confocal data suggested that most (all) rods make anatomical and electrical contact with a nearby cone, and that there is no color specificity in rod/cone connectivity. Most importantly, the confocal analysis placed the rod/cone gap junction within 1 µm of the opening of the post-synaptic compartment at the rod spherule, thus signposting the rod/cone gap junction, an observation that has proved useful to analyze SBF-SEM data (see below).

### SBF-SEM

**Figure 2:**
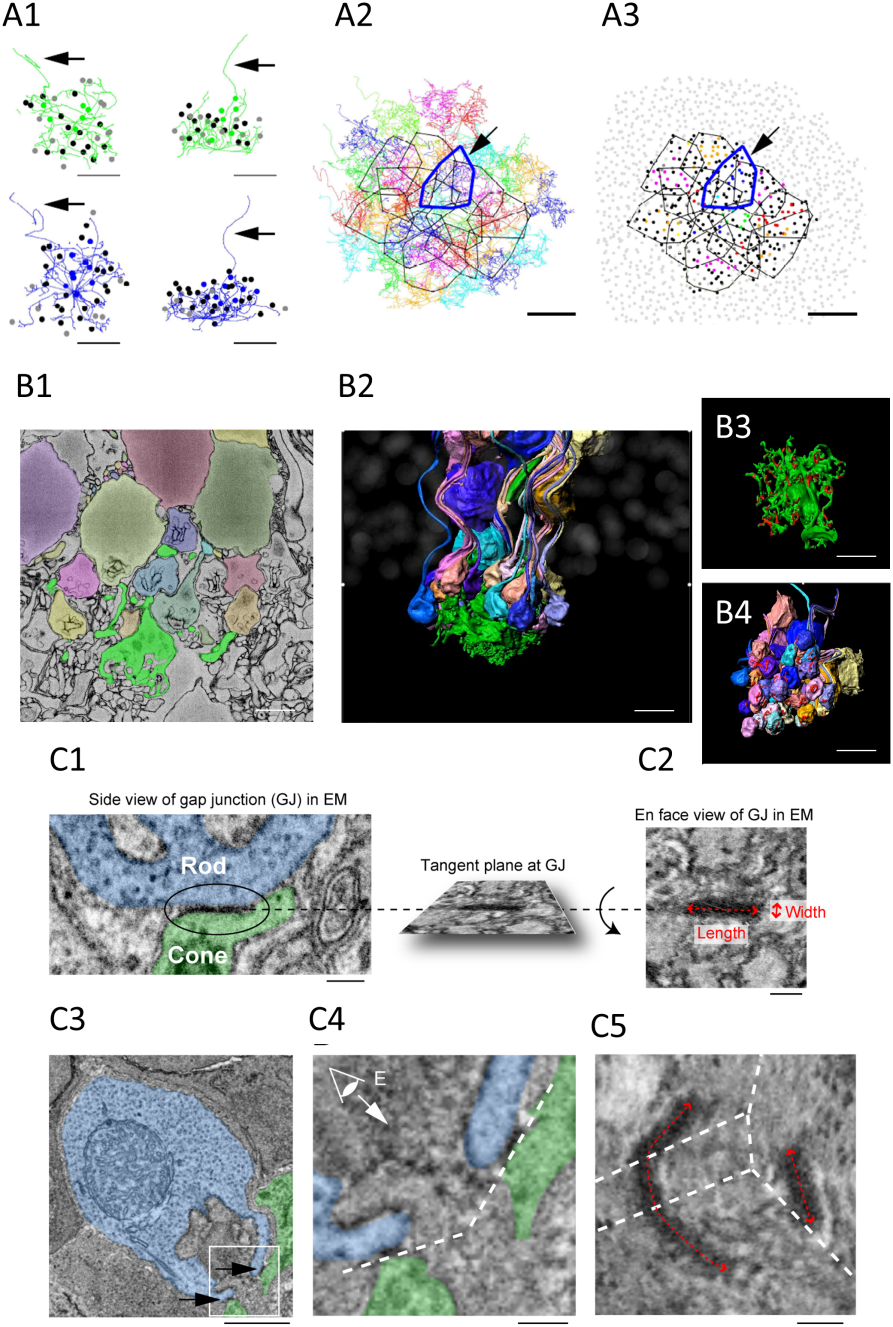
**(A1–3)** Cone skeleton analysis shows that cones contact all the nearby rod spherules. **(A1)** Skeletons of one green cone (green) and one blue cone (blue) in the wholemount view and projected. The black arrows show ascending axons. The position of each contacted rod spherule is marked by a dot, which is color coded the same color (green or blue) if the contacts are exclusive to this cone pedicle; black represents two cone contacts; and gray represents three cone contacts. Scale bar, 1 μm—applies to all. **(A2)** The telodendrial fields of 29 reconstructed cone pedicles, each have been color coded; the central 13 are outlined by polygons, and the arrow points to a blue cone (cone 2, thick blue outline). Cones 3 and 5 are also outlined with cyan and green, respectively. Scale bar, 5 μm. **(A3)** Outlines of the central 13 cone pedicles showing all rod spherule contacts. Each have been color coded and are exclusive to one cone; black represents contacts with two cones; and dark gray represents contacts with three cones. The light gray represents rod spherules outside the range of the central 13 cone pedicles. The arrow points to a blue cone (thick blue outline). Scale bar, 10 μm. **(B1–4)** Segmentation and 3D reconstruction from e2006. **(B1)** A single section showing a cone pedicle in green and contacted rods in various colors. Scale bar, 5 μm. **(B2)** Three-dimensional reconstruction of one cone pedicle (green) and all connected rods. **(B3)** The rotated view showing the top surface of the cone pedicle with contact pads in red. Scale bar, 5 μm. **(B4)** The rotated view— the bottom surface of the rod spherules with contact pads in red. Scale bar, 5 μm. **(C1–5)** The measurement of the gap junction size using focused ion beam-scanning electron microscopy (FIB-SEM). **(C1, 2)**. Electron-dense gap junction staining at a contact point between a cone telodendria (green) and a rod spherule (blue). The inset shows the rotated *en face* view of one gap junction used to measure the length and width. Scale bar, 0.2 μm. **(C3)** The image plane of a rod spherule (blue) through the synaptic opening with a small gap junction on each side (arrows) at two cone contacts (green). Scale bar, 1 μm. **(C4)** The enlarged picture of the inset in **(C3)** The dashed lines indicate tangent planes at gap junctions. **(C5)** To visualize one of the two gap junctions requires several planes. The dashed lines indicate intersections of the planes. The size of the right gap junction captured in one plane was measured as in **(C2)**. The length of the left gap junction was measured as the summed length from three planes. Scale bar, 0.2 μm. All figures and their respective descriptions are from Ishibashi et al. (24), with modifications.

SBF-SEM was useful to establish the rules of connectivity from large EM datasets. The distribution of Cx36 immunosignal at rod/cone contacts signposts the location of rod/cone gap junctions. Yet, a key limitation of confocal microscopy is the resolution—≈ 125 nm, at best, with Airyscan. This level of resolution prevents important aspects of the connectivity (e.g., rod-to-cone divergence) from being ascertained. To gain access to additional morphological details, Ishibashi et al. mapped the e2006 SBF-SEM dataset, which is derived from a block of mouse retina and is publicly available (73). A block of 29 adjacent cones was randomly chosen, at a resolution (voxel size) of 16.5 nm × 16.5 nm × 25 nm. These 29 cones showed a dense matrix of overlapping telodendria that connected 811 rods. The potential gap junctional contacts between the cone pedicles and rod spherules were established by following the telodendritic processes using skeletonization (**Figure 2A**). The skeleton data provided three important pieces of information: (1) each cone contacts every rod spherule within its telodendrial field (the convergence or number of rod spherule contacts per cone is about 43.0); (2) each rod spherule contacts more than one cone, usually two or three (the divergence or number of cones that connect a single rod is about 1.89); and (3) the blue (S) cones connectivity pattern with rods is similar to that of the green (M) cones.

Additionally, SBF-SEM analysis revealed that rods contact cones at various sites. The telodendritic tips and the roof of cone pedicles are the main sites. In addition, the lowest (most proximal) row of ONL rods, which do not have axons or spherules but do express synaptic machinery in the lowest part of their cell body, receive contacts from cone telodendria. Finally, some rod spherules are located below the cone pedicles and appear to have “missed” making a direct contact with the telodendria. These spherules are inverted and make contact with more proximal teledendria. As a rule, the cone contacts are always observed at the rod synaptic opening, corresponding with the position of Cx36. These locations therefore most likely have Cx36 rod/cone gap junctions.

The segmentation and 3D reconstruction of cone pedicles and connected rod spherules illustrate the complex field of telodendria extending laterally and upward (distally) from the pedicles, and the obvious position of the contacts, very close to the mouth of the synaptic invagination (**Figure 2B**). The cone contact sites reinforce the probable existence of rod/cone gap junctions, as they are consistent with the location of Cx36 clusters, as deduced from the confocal analysis. This is also supported by the fact that the average number of contact pads calculated from the full reconstruction of three cone pedicles was 56.7, on average, which is close to the number of Cx36 clusters per cone pedicle (51.4) which was calculated from the confocal analysis.

### FIB-SEM

FIB-SEM allowed visualization and measurement of rod/cone gap junctions. The SBF-SEM data provided important information about the general pattern of connectivity between the cones and rods. Yet, because of the relatively low resolution and heavy staining of the membranes, this approach was not appropriate to resolve definitive gap junctions in the tissue, although it is legitimate to assume that gap junctions are included in the contact pads. FIB-SEM provided isotropic data (same resolution in each dimension) at a 4-nm resolution and was used to search for gap junctions at rod/cone contacts. Areas of electron-dense staining, which are characteristic of gap junctions, were found in the same position as Cx36 labeling, as determined by confocal microscopy. In addition, the isotropic dataset allowed the rotation of the gap junctions and the estimation of their size from “en face” views (**Figure 2C**). This revealed the elongated form and orientation of rod/cone gap junctions encircling the opening of the synaptic opening of the rod spherule, in the same position as Cx36 labeling, as determined by confocal microscopy. The area of the gap junctions was typically smaller than that of contact pads measured in the SBF-SEM e2006 images and varied more in length (0.477 μm) than in width (0.123 μm), which is consistent with the string arrangement described by Raviola and Gilula (35). The number of gap junctions/rod spherule was found to be about 3.21. This number compares well with the number of Cx36 clusters observed via confocal microscopy (2.48). Our calculations below are based on the assumption that the rod/cone gap junctions in mice are arranged as strings of one-channel thick, as described in macaque and rabbit retinas (35). This is further supported by our measurements that showed that the length of rod/cone gap junctions is rather variable, whereas their width is rather constant. More recent observations from our laboratories further support the idea that mouse rod/cone gap junctions are strings. The fluorescence intensity of Cx36 clusters is about eight times dimmer, on average, in the OPL than in sublamina b of the IPL where Cx36 gap junction channels are known to be aggregated in large crystallin plaques of about 300 channels (3). This suggests that rod/cone gap junctions contain about 40 channels (300/8; 74). In addition, super-resolution microscopy revealed that rod/cone gap junctions are elongated structures, which is consistent with strings (74). Finally, in the newly developed phosphosphomimetic Cx36 mutant mouse (75), we found normal levels of expression of Cx36 at rod/cone contacts, yet measured rod/cone conductance close to 1,200 pS (O'Brien and Ribelayga unpublished observations). This shows that saturated rod/cone coupling is close to the theoretical maximum set by the morphological data and our calculations that are based on string arrangement (see below). Overall, these lines of evidence support the view that mouse rod/cone gap junctions are string-like structures of 40–50 channels long.

To fully comprehend the function of gap junctions in a circuit, determining the coupling strength and the location of gap junctions is an essential step. The number of gap junctions (N) and the conductance at each of these junctions (gj) determine the coupling conductance or coupling strength (Gc) between two electrically coupled neurons, that is:


(Equation 1) Gc=N*gj

In turn, gj depends on the number of connexons (n), the unitary conductance of the channel (γ), and the open probability, or percentage of open channels (Po) according to the equation:


(Equation 2) gj=n*γ*Po.

The morphological analysis outlined above can be used to calculate the mean theoretical maximum conductance between a rod/cone pair. The reconstructions of the mouse photoreceptor network indicated that every cone is coupled to nearby rods, with about 43 rods coupled to each cone (convergence). Each rod contacts about 1.89 cones on average (divergence). The average number of gap junctions at each rod spherule is about 3.2. Corrected for the divergence, this yields 1.7 (3.2/1.89) gap junctions between each rod/cone pair (N in Equation 1). The FIB-SEM data revealed that the mean length of a rod/cone gap junction is 480 nm. Assuming channel center-to-center spacing around 10 nm, we can calculate that a single rod/cone gap junction contains about 48 channels (480/10, n in Equation 2), a value that is close to the direct freeze-fracture EM measurements (≈ 50) reported by Raviola and Gilula (35). With Cx36 unitary conductance (γ in Equation 2) ≈ 15 pS (76; 77) and Po = 1 (i.e., all Cx36 channels between the pair are in an open state), we can calculate the mean maximal coupling conductance between a rod and a cone pair as:


(Equation 3) Gc=N * n * γ* Po=1.7 * 48 * 15 * 1≈1,200 pS.

Taking into account cumulative errors in our calculations yields a mean maximal coupling conductance of 1,228 ± 120 pS (mean ± SE) (see 24, for details).

The rod/cone coupling conductance has a resting value of about 300 pS (dark-adapted C57Bl/6J mouse), according to our recent data from paired recordings (23). Comparable to the value of 18% for Cx36 gap junctions in the cerebellum, where the precise number of connexons was determined by freeze fracture (15), this shows a resting open probability of 25% relative to the theoretical maximum of 1,200 pS (300/1,200). These findings surpass the conventional estimates of the open Cx36 channel fraction, which ranged between 0.1% and 1% (71, 72). In the presence of a D_2_-like dopamine receptor agonist (quinpirole), the rod/cone coupling conductance was decreased to about 50 pS (near the detection threshold), whereas an antagonist (spiperone) increased coupling to ≈ 1,200 pS (23). When compared with the theoretical maximum above, these statistics translate to a minimum open probability of 4% and a maximum close to 100% of the possible Cx36 channels (107% ± 10.6% with cumulative errors) (mean ± SE) (see 24 for details). Thus, the findings support a functional dynamic range of rod/cone coupling of approximately 24-fold (1,200/50) and suggest that every gap junction channel can participate when the coupling conductance equals the upper end of the operating range. The possibility for rod/cone gap junctions to recruit 100% of their channels is an astonishing result that uncovers a new property of electrical synapses and underlies the adaptive capabilities of neuronal networks in the retina. The dynamic range of this plasticity suggests that the dynamic modulation of signal transmission, facilitating learning, memory, and the fine-tuning of neural circuitry supported by electrical synapses in many other areas of the brain might be of a much larger amplitude than currently thought. In other words, this research may have uncovered a general property of electrical synapses with crucial functional significance.

## Conclusions and perspectives

Within the past decade, our collaborative work within the Vision research group, led by Steve Massey at the University of Texas Health Science Center at Houston, resulted in significant contributions to our understanding of the morphological basis and dynamic modulation of electrical coupling between photoreceptors. Steve has been instrumental in the success of this enterprise. We have now started to interrogate the function of rod/cone coupling in the visual system. In mammals, the rod/cone gap junction is the entry of an alternate pathway (i.e., the secondary rod pathway), through which rod signals are transmitted to retinal ganglion cells (RGCs) (78). The rod- or cone-specific Cx36 cKO lines, which lack functional rod/cone coupling, offer new tools to study the contribution of the secondary rod pathway to the retinal output and visual behavior. In fact, recent data showed a reduced contribution of rod signals to the light-adaptive process of the photopic ERG (79), to the light responses of OFF alpha RGCs (80), and to the pupillary light reflex (81), in the mutant lines. In that context, it is not unthinkable that the daily plasticity of rod/cone coupling impacts signal processing in the secondary rod pathway and thereby most, if not all, aspects of vision. Testing this possibility will be a primary task for future studies.

Our collaborative work has arguably positioned the rod/cone gap junction as a prime example of an electrical synapse in the CNS, of which we know the precise location, operating range, and for which we have the tools to interrogate its function. However, in most areas of the CNS there is still much we do not know about the size, distribution, and plasticity of gap junctions, despite the recent progress in our understanding of the molecular composition and cellular arrangement of gap junctions, and the discovery of the variety of disorders associated with connexins (82–84). The mapping of gap junctions in large EM datasets remains a major roadblock because of their small size and because most EM datasets are prepared to assess the morphology and connectivity of individual cells whose membranes are heavily stained for that very purpose. Combining immunolabeling for Cx36, high-resolution 3D-EM, and paired patch-clamp recordings from coupled neurons proved the right approach to harnessing the knowledge of rod/cone gap junction biology. As Cx36 is the primary neuronal connexin in the brain, the same approach should also be useful to interrogate the structure and function of gap junctions elsewhere in the CNS. The attractive possibility that all channels at Cx36 gap junctions may contribute to coupling should also motivate studies in this direction not only with the goal to gain new insights into their role in basic neuronal communication, but also with the objective of revisiting previous findings.

## Author contributions

CR: Funding acquisition, Writing – original draft, Writing – review and editing. JO: Funding acquisition, Writing – original draft, Writing – review & editing.
